# Educational attainment and cardiovascular disease in the United States: A quasi-experimental instrumental variables analysis

**DOI:** 10.1371/journal.pmed.1002834

**Published:** 2019-06-25

**Authors:** Rita Hamad, Thu T. Nguyen, Jay Bhattacharya, M. Maria Glymour, David H. Rehkopf

**Affiliations:** 1 Philip R. Lee Institute for Health Policy Studies, Department of Family & Community Medicine, University of California San Francisco, San Francisco, California, United States of America; 2 Department of Epidemiology & Biostatistics, University of California San Francisco, San Francisco, California, United States of America; 3 Department of Medicine, Stanford University, Stanford, California, United States of America; University of Oxford, UNITED KINGDOM

## Abstract

**Background:**

There is ongoing debate about whether education or socioeconomic status (SES) should be inputs into cardiovascular disease (CVD) prediction algorithms and clinical risk adjustment models. It is also unclear whether intervening on education will affect CVD, in part because there is controversy regarding whether education is a determinant of CVD or merely correlated due to confounding or reverse causation. We took advantage of a natural experiment to estimate the population-level effects of educational attainment on CVD and related risk factors.

**Methods and findings:**

We took advantage of variation in United States state-level compulsory schooling laws (CSLs), a natural experiment that was associated with geographic and temporal differences in the minimum number of years that children were required to attend school. We linked census data on educational attainment (*N* = approximately 5.4 million) during childhood with outcomes in adulthood, using cohort data from the 1992–2012 waves of the Health and Retirement Study (HRS; *N* = 30,853) and serial cross-sectional data from 1971–2012 waves of the National Health and Nutrition Examination Survey (NHANES; *N* = 44,732). We examined self-reported CVD outcomes and related risk factors, as well as relevant serum biomarkers. Using instrumental variables (IV) analysis, we found that increased educational attainment was associated with reduced smoking (HRS β −0.036, 95%CI: −0.06, −0.02, *p* < 0.01; NHANES β −0.032, 95%CI: −0.05, −0.02, *p* < 0.01), depression (HRS β −0.049, 95%CI: −0.07, −0.03, *p* < 0.01), triglycerides (NHANES β −0.039, 95%CI: −0.06, −0.01, *p* < 0.01), and heart disease (HRS β −0.025, 95%CI: −0.04, −0.002, *p* = 0.01), and improvements in high-density lipoprotein (HDL) cholesterol (HRS β 1.50, 95%CI: 0.34, 2.49, *p* < 0.01; NHANES β 0.86, 95%CI: 0.32, 1.48, *p* < 0.01), but increased BMI (HRS β 0.20, 95%CI: 0.002, 0.40, *p* = 0.05; NHANES β 0.13, 95%CI: 0.01, 0.32, *p* = 0.05) and total cholesterol (HRS β 2.73, 95%CI: 0.09, 4.97, *p* = 0.03). While most findings were cross-validated across both data sets, they were not robust to the inclusion of state fixed effects. Limitations included residual confounding, use of self-reported outcomes for some analyses, and possibly limited generalizability to more recent cohorts.

**Conclusions:**

This study provides rigorous population-level estimates of the association of educational attainment with CVD. These findings may guide future implementation of interventions to address the social determinants of CVD and strengthen the argument for including educational attainment in prediction algorithms and primary prevention guidelines for CVD.

## Abstract 2

Rita Hamad and colleagues reveal the associations between educational attainment and CVD events in the United States.

## Author summary

**Why was this study done?:**

- Heart disease is a leading cause of mortality in the US, and clinicians are increasingly interested in addressing its social and economic determinants.
- Education is highly correlated with heart disease, but this may be because education and heart disease have common causes like parental socioeconomic position and genetic factors.
- Even if there is an effect, the mechanisms linking education and heart disease are unclear.

**What did the researchers do and find?:**

- This study leveraged a natural experiment—variation in US education policies—to examine the effects of education on heart disease and its risk factors.
- Increased education was consistently associated with improvements in several cardiovascular risk factors: smoking, high-density lipoprotein, and depression.
- Increased education was also associated with higher BMI and total cholesterol.

**What do these findings mean?:**

- Policies to improve educational attainment may result in reduced burden of heart disease at the population level.
- Future studies should consider programs to measure or address educational attainment in clinical and community settings.

## Introduction

Prior work has suggested that clinicians should incorporate patients’ educational attainment into clinical decision-making, and that patients’ educational attainment could improve the accuracy of clinical predictive models such as the Framingham risk score [1]. Indeed cardiovascular mortality is underestimated in individuals of low socioeconomic status (SES) using the Framingham score, reflecting its focus on biomedical rather than social risk factors [2]. The 2019 guidelines from the American College of Cardiology and American Heart Association and the US Department of Health and Human Services have suggested using patients’ social factors in clinical prediction tools and to risk-adjust physician panels in determining physician payments for performance [3,4]. Yet while numerous studies have linked low educational attainment to risk of cardiovascular disease (CVD), few provide population-level estimates, and many existing studies cannot rule out confounding by unmeasured factors such as genetic endowment or parental SES [5]. These obstacles pose challenges for rigorously estimating the impact of education on CVD, hindering the ability to implement appropriate interventions. A recent review concluded that there is substantial disagreement in the education-health literature due to confounding, warranting additional research on this topic [6].

**Fig 1:**
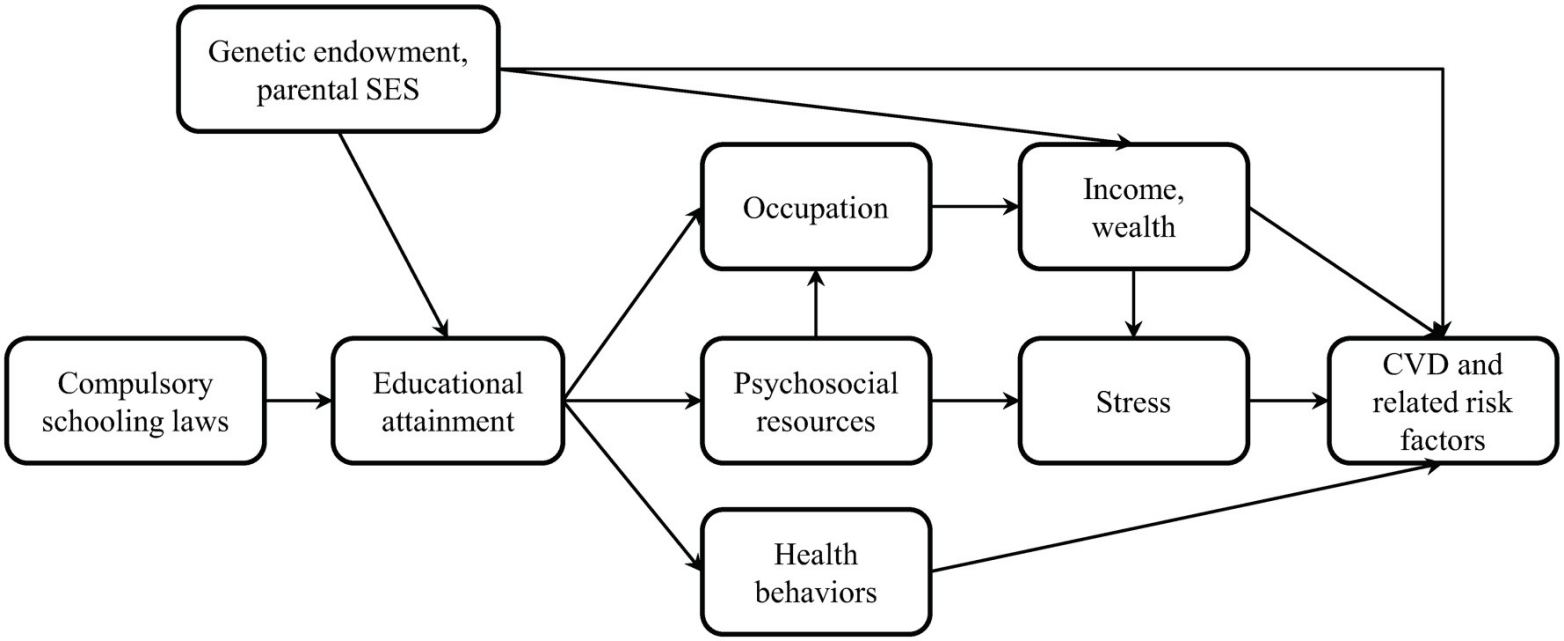
Conceptual model linking educational attainment and CVD. CVD, cardiovascular disease; SES, socioeconomic status.

There are numerous hypothesized pathways linking education with CVD (Fig 1). Increased educational duration, quality, and credentials are thought to increase employment [7]; augment psychosocial resources such as literacy, social capital, and decision-making [8–11]; and improve health behaviors like smoking [12,13]. Psychosocial resources and employment, in turn, may increase income and decrease stress. Each of these may then lead to reduced CVD.

Given challenges in implementing randomized trials in this field, studies increasingly apply “quasi-experimental” methods to examine the links between education and health [14], taking advantage of natural experiments such as expansions in Head Starts and other social policies [15]. Although several studies randomized children to high-quality early childhood interventions and demonstrated improved cardiovascular health later in life, sample sizes were small, with limited long-term follow-up [16], and randomization of public education is typically not feasible or ethical. Population-level estimates of the effects of education on CVD are largely lacking.

In this study, we took advantage of a natural experiment in the form of US compulsory schooling laws (CSLs), state policies that legislate the number of years children must attend school. CSLs create differences by state and across time in the duration of education [17]. Numerous studies have exploited this natural experiment to determine the impact of education on economic outcomes. Using CSLs as instrumental variables (IV) for educational attainment, these have found increased earnings and employment, and intergenerational impacts on social outcomes among children of those affected by CSLs [7,18,19]. A recent meta-analysis of studies that examined the health effects of CSLs highlighted the small number of US studies, which have focused primarily on the impact of education on mortality and fertility [20]. Drawing on data from several countries, this meta-analysis found improvements in smoking, obesity, and mortality but insufficient evidence for other outcomes. The examination of other outcomes is critical to understand the pathways through which education may influence CVD, as this would inform subsequent interventions to reduce CVD disparities. While numerous studies on CSLs and CVD and related risk factors have been conducted in Europe [21–25], findings may not generalize to the US due to political- and sociocultural-based differences in the role of education. Two published studies have examined the effects of CSLs on CVD in the US context, with one study finding reductions in self-reported heart attack and diabetes risk, and the other finding reductions in self-reported diabetes and hypertension but no effect for “heart trouble” [26,27]. To our knowledge, no published studies in the US have examined the effects of CSLs on objective biomarkers of CVD.

In this study, we leveraged a natural experiment to test the hypothesis that educational duration affects CVD outcomes and related risk factors, examining multiple pathways through which education may affect CVD. We linked administrative data on CSLs with two large nationally representative US data sets and employ the quasi-experimental method of IV analysis. In addition to estimating rigorous population-level effects of education on CVD, this study contributes evidence on a specific educational policy, thereby guiding future implementation of social and educational interventions to address the social determinants of CVD.

## Methods

### Data

This study involved the integration of several large data sets, with all analyses prespecified (see S1 Analytic Plan). As described below, we conducted a two-sample IV analysis. The first stage of the IV analysis was conducted among US-born individuals in the US Census 5% sample (*N* = approximately 5.4 million). We used the 1980 Census because demographic questions were comparable to and birth years of participants overlapped with those in the US Health and Retirement Study (HRS) and the National Health and Nutrition Examination Survey (NHANES), the data sets from which CVD outcomes were derived.

To estimate the second stage of the IV analysis, we linked first-stage census estimates with two data sets that included the outcomes of interest: HRS and NHANES. In other words, years of compulsory schooling and predicted years of educational attainment determined in census data were linked to each individual in HRS and NHANES based on his/her birth year, birth state, race, and sex. HRS is a longitudinal nationally representative US study of individuals age 50 or older and their spouses. The first survey wave was conducted in 1992, with biennial interviews subsequently. The second data set was NHANES, a serial cross-sectional US study conducted in 1971–1974 (NHANES I), 1976–1980 (NHANES II), 1988–1994 (NHANES III), and biennially since 1999. For both data sets, we included survey waves through 2012, the most recent data available at the onset of data analysis. We restricted the data sets to US-born individuals with data on state of birth and at least one CVD outcome. In NHANES, we also restricted the data set to white and black individuals, due to inconsistencies in categorization of other races/ethnicities across survey waves. Data on CSLs and state characteristics were compiled using federal reports for 1900–1950 [28], and health outcomes included markers of CVD more prevalent in adulthood; we therefore restricted the data set to individuals born during 1900–1950 who were at least 18 when surveyed.

Final sample sizes were 30,853 (HRS) and 44,732 (NHANES), although the number of observations was smaller for outcomes not obtained for all participants in all waves (Table 1).

**Table 1:** Participant characteristics.

Characteristics	HRS		NHANES I		NHANES II		NHANES III		NHANES 1999–2012	
	*N* = 30,853		*N* = 11,254		*N* = 11,218		*N* = 8,413		*N* = 13,847	
**Demographics**	Value		Value		Value		Value		Value	
Year of birth (mean ± SD)	1936 ± 14		1927 ± 17		1934 ± 20		1933 ± 18		1941 ± 14	
Race (%)										
White	76.2		82.9		87.5		65.2		74.8	
Black	17.4		17.1		12.5		34.8		25.2	
Hispanic	4.8									
Other	1.7									
Female (%)	55.7		60		52.1		54.6		50.6	
Education (%)										
Less than high school	23.6		46.5		41		37.8		24.8	
High school	35.2		31.9		32.1		32.1		27.2	
More than high school	41.2		21.5		26.9		30.2		48	
**Health Outcomes**	Value	Sample size	Value	Sample size	Value	Sample size	Value	Sample size	Value	Sample size
Self-reported										
Hypertension (%)	63.4	30,835	22	8,799	25.6	11,185	38	8,383	48.3	13,812
Diabetes (%)	24.5	30,828	4.3	8,799	4.1	11,185	10.1	8,405	14.5	13,542
Heart disease (%)	34.5	30,826								
Smoking (%)	60.2	30,661			35	9,904	25.9	8,402	20.1	13,834
Depression (%)	36.8	29,315								
Anthropometric										
BMI (mean ± SD)	27.5 ± 5.6	30,241	25.2 ± 5.4	11,248	25.0 ± 5.1	11,218	27.3 ± 6	7,459	29.0 ± 6.6	12,598
Blood pressure (mmHg, mean ± SD)	130.2 ± 20.8	7,950	131.8 ± 24	11,200	128.2 ± 22.8	11,165	131.2 ± 20.8	7,162	130.6 ± 21.3	12,418
Biomarkers^a^										
Hyperglycemia^b^ (%)	13.5	15,650			6.1	3,028	10.6	7,085	12.1	12,398
Total cholesterol (mg/dL, mean ± SD)	199.8 ± 42.7	15,515	216.6 ± 49.4	11,205	217.7 ± 49.4	9,398	213.4 ± 44.6	6,996	203.6 ± 42.5	12,237
HDL cholesterol (mg/dL, mean ± SD)	54.16 ± 16	13,672			49.7 ± 14.6	7,850	52.06 ± 16.9	6,945	54.2 ± 17.1	12,234
LDL cholesterol (mg/dL, mean ± SD)							133.9 ± 38.7	2,918	120.2 ± 37.3	5,647
Triglycerides (mg/dL, median [IQR])					122 (86–179)	4,564	82 (118–174)	6,980	85 (118–172)	5,974
CRP (mg/dL, median [IQR])	2.1 (1.0–4.6)	15,452					0.21 (0.21–0.5)	6,934	0.24 (0.1–0.54)	12,299
Telomere length (base pairs, median [IQR])	3,069 (2,679–3,566)	5,040							5,551 (5,211–5,951)	3,581

^a^For non-normally distributed outcomes, we display the median and IQR rather than mean and SD.

^b^For serum testing of diabetes, earlier waves of NHANES used 2-hour glucose testing, while later waves and HRS used hemoglobin A1c. For consistency, we transformed these into a binary measure of whether they exceeded the cutoff for diabetes (i.e., glucose ≥ 200, hemoglobin A1c ≥ 6.5).

Abbreviations: CRP, C-reactive protein; HDL, high-density lipoprotein; HRS, Health and Retirement Study; IQR, interquartile range; LDL, low-density lipoprotein; NHANES, National Health and Nutrition Examination Survey; SD, standard deviation.

### Predictor

The primary predictor in ordinary least squares (OLS) models was self-reported educational attainment (continuous in census and HRS, categorical in NHANES). This was also the dependent variable in the first stage of the IV analyses, described below.

### Outcomes

Outcomes included serum biomarkers, anthropometric measures, and self-reported outcomes of CVD and related risk factors previously correlated with education (Table 1). Each outcome represented one or more mechanistic pathways through which education might influence CVD. For example, diabetes and cholesterol in part reflect health behaviors such as nutrition and physical activity. Meanwhile, C-reactive protein (CRP) and telomere length measure inflammatory pathways and may capture chronic stress [29,30]. Studies suggest that socioeconomic disparities accelerate CVD by heightening stress responses [31,32]. Similarly, depression is a risk factor for mortality among patients with CVD [32] and was operationalized as a score of 3 or more on the shortened 8-item Center for Epidemiologic Studies Depression scale [33]. For outcomes that were heavily skewed and for which residuals were non-normally distributed—telomere length, CRP, and triglycerides—the natural logarithm was taken. Of note, higher levels of telomere length and high-density lipoprotein (HDL) and lower levels of other biomarkers are considered beneficial.

When possible, we chose outcomes that were similar across NHANES and HRS to cross-validate findings. For example, earlier waves of NHANES included 2-hour glucose testing, while later waves and HRS included hemoglobin A1c (also known as glycated or glycosylated hemoglobin). For consistency, we created a binary measure of whether the level exceeded the cutoff for diabetes (glucose ≥ 200, hemoglobin A1c ≥ 6.5). For CRP, HRS includes a variable for CRP that is constructed to be equivalent to that measured in NHANES [34]. Most self-reported outcomes included similar wording across both surveys, e.g., “Has a doctor ever told you that you have high blood pressure or hypertension?”

For some outcomes, however, parallel questions were not included in both HRS and NHANES, so we only included outcomes from a single data set. For example, NHANES did not include comparable questions on heart disease, and early waves did not include questions on depression, while HRS does not measure triglycerides. In HRS—which consists of repeated surveys of the same individuals over time—self-reported outcomes were coded as 1 if the respondent ever stated that they had the disease (parallel to NHANES question formats), and labs and anthropometric measures represent the first available value of the outcome to minimize survivorship bias.

### Covariates

We controlled for variables that may confound the relationship between exposure to CSL policies and CVD. These included race, gender, and birth year, as well as time-varying state-level characteristics to address potential state-level confounding. These included percentage black, urban, and foreign born; manufacturing jobs per capita; and inflation-adjusted manufacturing wages per manufacturing job. These were compiled from Statistical Abstracts of the US and linearly interpolated for years between reports [35], and have been similarly included as covariates in prior CSL studies [28].

### Data analysis

We first tabulated HRS and NHANES participant characteristics. We then conducted two sets of analyses: (1) OLS, which is subject to confounding of the relationship between educational attainment and CVD, and (2) IV, which is intended to address this confounding.

### OLS models

In OLS models, we regressed each outcome on self-reported educational attainment in HRS and NHANES. The primary predictor variable in HRS was a continuous variable for self-reported educational attainment in years. For NHANES, the primary predictor was the “more than high school” category of educational attainment (reference: less than high school), as NHANES does not include a continuous variable for education in all survey waves. A similar analysis was carried out in HRS using a categorical education variable, for comparability. We adjusted for individual- and state-level characteristics described above.

Because the treatment—that is, educational quality—is at the state level, we clustered standard errors by state [36] using the Huber-White heteroscedasticity-robust sandwich estimator to account for correlated observations [37]. Notably, early waves of NHANES employ Fay’s replicate weights for variance estimation [38], while later waves of NHANES include probability sampling weights. To our knowledge, there is no established method to pool surveys that incorporate these different techniques for sample weighting, so we were unable to incorporate sample weights in our analysis. Regardless, the appropriateness of sample weighting is diminished when the goal of analysis is estimation of treatment effects rather than producing descriptive population statistics [39], so this is unlikely to introduce bias into the results.

### IV models

OLS models suffer from confounding by unobserved individual factors like genetic endowment or parental SES. Therefore, we next carried out the quasi-experimental method of IV analysis, a well-established technique in epidemiology and clinical medicine [40]. As shown in S1 Fig, IV methods rely on the presence of a quasi-randomly determined exposure or “instrument” (Z)—in this case, CSLs—that is known to impact the predictor of interest (X, education). This perturbation in X caused by Z is then used to infer the effects of X on the relevant outcomes (Y). This method is particularly useful when X cannot be randomized, and when the relationship between X and Y may be confounded by unmeasured individual characteristics (U1) (see S1 Text for details).

In this study, the IV analysis leveraged the natural experiment created by CSLs to estimate effects of education that are unconfounded by unobserved individual factors. In particular, we employed two-sample IV analysis, in which the first and second stages were carried out in two different data sets [41,42]. Using a two-sample approach allowed for more precise estimation of the first stage, as the census sample size was much larger, thereby alleviating concerns of weak instrument bias resulting from instruments that explain only a small fraction of the variation in the endogenous variable [43]. Two-sample IV analysis is also useful in situations in which a single data set does not include information on all three variables of interest [44]—i.e., the outcome, the predictor (self-reported educational attainment), and the instrument (CSLs). In this case, early waves of NHANES did not include a continuous measure of educational attainment, highlighting the utility of the two-sample IV approach. Additional details on the two-sample IV analyses, including equations, are provided in S1 Text.

We used two IVs to capture the number of minimum years of compulsory schooling in an individual’s state of birth [17,18,28]. The first was the difference between compulsory enrollment age in the state of birth when the respondent was 6 and minimum dropout age when the respondent was 14, and the second was the difference between compulsory enrollment age when the respondent was 6 and minimum work age when the respondent was 14. We assumed that individuals remained in their state of birth until age 18; prior studies have shown that cross-state migration was low during this period and that it was uncorrelated to the implementation of CSLs, so any measurement error (i.e., misclassification) would likely bias our results to the null [17,45].

Robust standard errors were calculated using a bootstrapping technique, again clustered at the state level to account for correlated observations (see S1 Text for details).

### Fixed effects analyses

Prior work has shown that IV estimates of the effects of education may be sensitive to the inclusion of fixed effects (i.e., indicator variables) for state of birth [27,28], which control for unobserved time-invariant state-level confounders but reduce statistical power. We conducted an additional set of OLS and IV analyses that included state fixed effects. The Durban-Wu-Hausman test demonstrated that there were no systematic differences between OLS and fixed effects models (*p* > 0.05 for all outcomes) [46], and fewer than 5% of coefficients for state of birth were statistically significant. Thus, we have little empirical evidence that state of birth was a confounder in these analyses. Nevertheless, we present fixed effects models alongside OLS models, given that state-level characteristics may still be considered confounders on theoretical grounds, although it should be noted that these models reduce power and the amount of variation in the exposure because fixed effects models only leverage variation in the exposure within rather than between states.

### Missing values

Less than 3% of covariates were missing. Complete case analysis is unlikely to introduce bias at such low levels of missingness [47–50]. We did not impute missing outcomes, as this is thought to add noise to subsequent estimates [51].

### Multiple hypothesis testing

To account for the examination of multiple outcomes, we calculated adjusted *p*-values using the Dubey/Armitage-Parmar method, a modification of the Bonferroni method that accommodates correlated outcomes [52,53].

### Ethics approval

Ethics approval was provided by the University of California, San Francisco (#17–21575). Approval for HRS was provided by the University of Michigan; approval for NHANES was provided by the National Center for Health Statistics.

## Results

### Participant characteristics

Participant characteristics were similar across HRS and NHANES, with slightly over half of the participants female and about three quarters white (Table 1). About two thirds of individuals had completed high school education or less. CVD measures were generally worse in HRS, which includes older individuals than NHANES. Because most of the outcomes were obtained using similar questions and laboratory methods, these differences therefore likely represent age and cohort effects, which we account for in our models by adjusting for birth year.

### OLS models

Higher educational attainment was associated with improvements for all outcomes except total cholesterol (Table 2). Except for telomere length in HRS, all of these associations were robust to the adjustment of *p*-values for multiple hypothesis testing. Coefficients for NHANES were roughly comparable to those in HRS when using a comparable categorical variable for education as the primary exposure, although the HRS estimate for telomere length was no longer statistically significant at *p* < 0.05, likely due to the conversion of the primary predictor from continuous to categorical.

**Table 2:** Association of self-reported educational attainment with CVD and risk factors (OLS analysis).

Outcome	HRS		NHANES
	Coefficient for 1 Year of Education	Coefficient for More than High School^a^	Coefficient for More than High School^a^
**Self-reported**			
Hypertension	−0.0083**	−0.060**	−0.049**
	(−0.010 to −0.0065)	(−0.073 to −0.047)	(−0.060 to −0.038)
Diabetes	−0.010**	−0.081**	−0.043**
	(−0.013 to −0.0082)	(−0.097 to −0.066)	(−0.051 to −0.035)
Heart disease	−0.0096**	−0.068**	
	(−0.011 to −0.0080)	(−0.082 to −0.054)	
Smoking	−0.017**	−0.12**	−0.17**
	(−0.020 to −0.014)	(−0.13 to −0.100)	(−0.18 to −0.15)
Depression	−0.027**	−0.19**	
	(−0.029 to −0.024)	(−0.21 to −0.18)	
**Anthropometric**			
BMI	−0.12**	−0.86**	−0.23**
	(−0.15 to −0.099)	(−0.99 to −0.72)	(−0.41 to −0.063)
Blood pressure	−0.64**	−4.10**	−2.51**
	(−0.81 to −0.46)	(−5.23 to −2.97)	(−3.07 to −1.96)
**Biomarkers**			
Hyperglycemia	−0.0073**	−0.055**	−0.051**
	(−0.0096 to −0.0049)	(−0.074 to −0.035)	(−0.064 to −0.039)
Total cholesterol	0.15	0.56	0.70
	(−0.15–0.45)	(−1.78–2.90)	(−0.49–1.90)
HDL cholesterol	0.47**	2.54**	2.58**
	(0.35–0.59)	(1.76–3.31)	(2.12–3.04)
LDL cholesterol			−2.2
			(−4.64–0.24)
Ln(Triglycerides)			−0.11**
			(−0.13 to −0.090)
Ln(CRP)	−0.049**	−0.29**	−0.20**
	(−0.056 to −0.042)	(−0.34 to −0.25)	(−0.24 to −0.15)
Ln(Telomere length)	0.0031*	0.017	0.013**
	(0.00046–0.0058)	(−0.0055–0.039)	(0.0054–0.021)

^a^ Reference: less than high school.

* *p* < 0.05.

** *p* < 0.01.

Confidence interval (95%) in parentheses. The primary predictor variable in HRS is a continuous variable for self-reported educational attainment in years. For NHANES, the primary predictor is the “more than high school” category of educational attainment (reference: less than high school), as NHANES does not include a continuous variable for education in all survey waves. A similar analysis with a categorical exposure variable was carried out in HRS for comparability. Analyses involved linear models using OLS, with robust standard errors clustered by state. All models adjust for individual-level gender, race, and year of birth and state-level percent urban, percent foreign born, percent black, manufacturing wages, and manufacturing jobs per capita.

Abbreviations: CRP, C-reactive protein; CVD, cardiovascular disease; HDL, high-density lipoprotein; HRS, Health and Retirement Study; LDL, low-density lipoprotein; NHANES, National Health and Nutrition Examination Survey; OLS, ordinary least squares.

### IV models

The F statistic for the first stage of IV models using census data was 793.7. This was above the standard cutoff of 10, indicating that CSLs are a strong instrument for education [54].

In HRS (Table 3), increased education was associated with reduced heart disease (β −0.025; 95%CI: −0.04, −0.002; *p* = 0.01), smoking (β −0.036; 95%CI: −0.06, −0.02; *p* < 0.01), and depression (β −0.049; 95%CI: −0.07, −0.03; *p* < 0.01); improved HDL (β 1.50; 95%CI: 0.34, 2.49; *p* < 0.01); and worsened total cholesterol (β 2.73; 95%CI: 0.09, 4.97; *p* = 0.03) and BMI (β 0.20; 95%CI: 0.002, 0.40; *p* = 0.05). The estimates for smoking, depression, and HDL were robust to the adjustment of *p*-values for multiple hypothesis testing.

**Table 3:** Effect of educational attainment on CVD and risk factors (IV analysis).

Outcome	HRS	NHANES
	Effect of 1 Year of Education	
**Self-reported**		
Hypertension	−0.015	−0.0033
	(−0.04–0.01)	(−0.02–0.01)
Diabetes	−0.011	0.0031
	(−0.03–0.01)	(−0.01–0.02)
Heart disease	−0.025*	
	(−0.04 to −0.002)	
Smoking	−0.036**	−0.032**
	(−0.06 to −0.02)	(−0.05 to −0.02)
Depression	−0.049**	
	(−0.07 to −0.03)	
**Anthropometric**		
BMI	0.20*	0.13*
	(0.002–0.40)	(0.01–0.32)
Blood pressure	−1.25	−0.11
	(−3.01–0.51)	(−0.68–0.69)
**Biomarkers**		
Hyperglycemia	−0.0055	0.0031
	(−0.03–0.01)	(−0.01–0.02)
Total cholesterol	2.73*	−0.46
	(0.09–4.97)	(−2.04–1.04)
HDL cholesterol	1.50**	0.86**
	(0.34–2.49)	(0.32–1.48)
LDL cholesterol		−1.44
		(−4.47–1.26)
Ln(Triglycerides)		−0.039**
		(−0.06 to −0.01)
Ln(CRP)	−0.055	−0.025
	(−0.13–0.01)	(−0.07–0.03)
Ln(Telomere length)	−0.010	−0.0054
	(−0.04–0.02)	(−0.02–0.01)

*p < 0.05.

**p < 0.01.

Confidence interval (95%) in parentheses. Analyses involved two-sample IV analyses, with the first stage conducted in the 1980 5% Census sample. All models adjust for individual-level gender, race, and year of birth and state-level percent urban, percent foreign born, percent black, manufacturing wages, and manufacturing jobs per capita. Standard errors were calculated using 10,000 bootstrap samples.

Abbreviations: CRP, C-reactive protein; CVD, cardiovascular disease; HDL, high-density lipoprotein; HRS, Health and Retirement Study; IV, instrumental variable; LDL, low-density lipoprotein; NHANES, National Health and Nutrition Examination Survey.

In NHANES, increased education was associated with reduced smoking (β −0.032; 95%CI: −0.05, −0.02; *p* < 0.01) and triglycerides (β −0.039; 95%CI: −0.06, −0.01; *p* < 0.01) and improved HDL (β 0.86; 95%CI: 0.32, 1.48; *p* < 0.01), but higher BMI (β 0.13; 95%CI: 0.01, 0.32; *p* = 0.05). Each of these except for BMI was robust to the adjustment of *p*-values for multiple hypothesis testing.

### Fixed effects models

When adjusting for state fixed effects, OLS estimates were similar to models without fixed effects in both HRS and NHANES (Table 4), with improvements in all outcomes except for total and low-density lipoprotein (LDL) cholesterol. Except for telomere length in HRS, all of these associations were robust to the adjustment of *p*-values for multiple hypothesis testing. As in OLS models without fixed effects, coefficients for NHANES were roughly comparable to those in HRS when using a comparable categorical variable for education as the primary exposure, although the HRS estimate for telomere length was again no longer statistically significant at *p* < 0.05, likely due to the conversion of the primary predictor from continuous to categorical.

**Table 4:** Association of self-reported educational attainment with CVD and risk factors, with state fixed effects (OLS analysis).

Outcome	HRS		NHANES
	Coefficient for 1 Year of Education	Coefficient for More than High School^a^	Coefficient for More than High School^a^
**Self-reported**			
Hypertension	−0.0083**	−0.058**	−0.048**
	(−0.010 to −0.0064)	(−0.072 to −0.044)	(−0.059 to −0.037)
Diabetes	−0.010**	−0.080**	−0.042**
	(−0.013 to −0.0082)	(−0.095 to −0.065)	(−0.050 to −0.034)
Heart disease	−0.0094**	−0.066**	
	(−0.011 to −0.0078)	(−0.080 to −0.052)	
Smoking	−0.017**	−0.12**	−0.17**
	(−0.020 to −0.014)	(−0.13 to −0.100)	(−0.18 to −0.15)
Depression	−0.027**	−0.19**	
	(−0.029 to −0.024)	(−0.21 to −0.17)	
**Anthropometric**			
BMI	−0.12**	−0.84**	−0.26**
	(−0.15 to −0.099)	(−0.98 to −0.71)	(−0.43 to −0.084)
Blood pressure	−0.63**	−4.00**	−2.47**
	(−0.80 to −0.46)	(−5.11 to −2.89)	(−3.01 to −1.92)
**Biomarkers**			
Hyperglycemia	−0.0071**	−0.053**	−0.050**
	(−0.0094 to −0.0049)	(−0.073 to −0.034)	(−0.063 to −0.038)
Total cholesterol	0.12	0.37	0.56
	(−0.19–0.43)	(−2.00–2.73)	(−0.61–1.72)
HDL cholesterol	0.45**	2.39**	2.48**
	(0.32–0.57)	(1.62–3.17)	(2.01–2.95)
LDL cholesterol			−1.90
			(−4.31–0.52)
Ln(Triglycerides)			−0.11**
			(−0.13 to −0.087)
Ln(CRP)	−0.049**	−0.29**	−0.20**
	(−0.056 to −0.041)	(−0.34 to −0.24)	(−0.24 to −0.15)
Ln(Telomere length)	0.0036*	0.019	0.014**
	(0.00071–0.0065)	(−0.0043–0.043)	(0.0077–0.021)

^a^Reference: less than high school.

*p < 0.05.

**p < 0.01.

Confidence interval (95%) in parentheses. The primary predictor variable in HRS is a continuous variable for self-reported educational attainment in years. For NHANES, the primary predictor is the “more than high school” category of educational attainment (reference: less than high school), as NHANES does not include a continuous variable for education in all survey waves. A similar analysis with a categorical exposure variable was carried out in HRS for comparability. Analyses involved linear models using OLS, with robust standard errors clustered by state. All models adjust for individual-level gender, race, and year of birth and state-level percent urban, percent foreign born, percent black, manufacturing wages, and manufacturing jobs per capita.

Abbreviations: CRP, C-reactive protein; CVD, cardiovascular disease; HDL, high-density lipoprotein; HRS, Health and Retirement Study; LDL, low-density lipoprotein; NHANES, National Health and Nutrition Examination Survey; OLS, ordinary least squares.

For IV analyses (Table 5), in HRS the confidence intervals for each estimate included the null, although all (including total cholesterol) had point estimates suggesting improvement. When adjusting IV models for state fixed effects in NHANES, all confidence intervals included the null, and there was no consistent direction of effect estimates.

**Table 5:** Effect of educational attainment on CVD and risk factors, with state fixed effects (IV analysis).

Outcome	HRS	NHANES
	Effect of 1 Year of Education	
**Self-reported**		
Hypertension	0.032	−0.0087
	(−0.12–0.16)	(−0.1–0.1)
Diabetes	0.017	−0.00020
	(−0.12–0.17)	(−0.06–0.07)
Heart disease	−0.029	
	(−0.17–0.12)	
Smoking	−0.13	0.11
	(−0.28–0.01)	(0–0.23)
Depression	−0.14	
	(−0.3–0.03)	
**Anthropometric**		
BMI	−0.21	−0.11
	(−1.71–1.70)	(−1.17–0.98)
Blood pressure	6.08	−1.88
	(−12.2–24.6)	(−5.57–3.36)
**Biomarkers**		
Hyperglycemia	0.086	0.016
	(−0.09–0.24)	(−0.08–0.12)
Total cholesterol	−12.03	−2.22
	(−31.48–10.72)	(−11.88–8.55)
HDL cholesterol	3.71	−0.60
	(−4.99–12.68)	(−6.65–4.31)
LDL cholesterol		−4.47
		(−31.46–22.04)
Ln(Triglycerides)		0.22
		(0–0.47)
Ln(CRP)	0.41	−0.16
	(−0.18–0.99)	(−0.61–0.28)
Ln(Telomere length)	0.082	−0.021
	(−0.21–0.36)	(−0.1–0.06)

*p < 0.05.

**p < 0.01.

Confidence interval (95%) in parentheses. Analyses involved two-sample IV analyses, with the first stage conducted in the 1980 5% Census sample. All models adjust for individual-level gender, race, and year of birth and state-level percent urban, percent foreign born, percent black, manufacturing wages, and manufacturing jobs per capita. Standard errors were calculated using 10,000 bootstrap samples.

Abbreviations: CRP, C-reactive protein; CVD, cardiovascular disease; HDL, high-density lipoprotein; HRS, Health and Retirement Study; IV, instrumental variable; LDL, low-density lipoprotein; NHANES, National Health and Nutrition Examination Survey.

## Discussion

### Summary

In this study, we exploited a natural experiment—variation in US CSLs—to estimate the effects of educational attainment on CVD in later life, examining several outcomes to better understand the different pathways through which education may influence CVD. This study provides population-level estimates of the effects of education on CVD for inclusion in clinical prediction and risk adjustment models. It also provides more rigorous estimates of the causal effect of educational attainment on CVD to inform future interventions to address this important social determinant, because correlational estimates like our OLS models may suffer from confounding or reverse causation. While the OLS models suggested improvements in virtually all health outcomes, IV models suggested improvements only for smoking, depression, heart disease, and HDL, and possibly worsened total cholesterol and BMI.

Overall, the evidence from HRS indicates that education is associated with reduced heart disease. Based on our exploration of pathways below, this may be driven by improvements in smoking, HDL, and depression. These findings suggest that the relationship between education and CVD risk factors may be causal, making it a potentially important predictor to target in clinical and policy interventions. Unfortunately, the self-reported measure of heart disease in HRS does not specify the type of medical condition included in “heart disease”—e.g., myocardial infarction, heart failure, or atrial fibrillation—which limits our ability to fully understand the pathways at play.

Our results also highlight the importance of incorporating social determinants into clinical prediction algorithms. For example, prior work has found that incorporating a marker of neighborhood-level social deprivation into a cardiovascular risk score greatly reduced socioeconomic disparities in identification of disease relative to the Framingham score; including neighborhood deprivation in CVD risk algorithms is therefore increasingly incorporated into guidelines in numerous international settings [55–57]. Yet there remains controversy over the incorporation of education into risk adjustment algorithms. For example, adjusting physician payment based on the distribution of social factors in their patient panels may encourage physicians to care for low-SES patients, because they will not be penalized for the generally worse outcomes that occur in this group of patients. Yet, it may lead to decreased quality of care and potentially increased disparities for low-SES patients, because standards of care will be different (i.e., probably lower) for these groups [3]. On the other hand, not risk-adjusting may lead physicians to cherry-pick higher-SES (and likely healthier) patients to meet performance guidelines, thereby worsening disparities [58].

### Insight into mechanisms

In terms of the mechanisms linking education and CVD, correlational OLS models suggested improvements in virtually all outcomes, yet IV models found that education was associated with only a handful, including worsening of some risk factors. Two of these—reduced smoking and improved HDL cholesterol—were observed in both HRS and NHANES. A recent meta-analysis of international CSLs also found improvements in smoking [20]; no prior study to our knowledge has examined the effects of CSLs on HDL. As HDL is linked to physical activity, education may influence HDL by increasing physical activity. Alternately, these improvements may be due to improved medical care, because education is known to increase employment opportunities [7,18], and in the US employment is linked to health insurance and healthcare access. We were unable to reject the null hypothesis that there was no benefit for most outcomes linked to nutrition or healthcare access (e.g., LDL cholesterol, hyperglycemia); wide confidence intervals suggest that these analyses were underpowered, because there were fewer individuals who participated in biomarker testing.

In contrast, education was associated with increased total cholesterol in HRS and increased BMI in both samples, which may represent the health behavior pathway. This contradicts findings from a recent meta-analysis suggesting that CSLs in international settings lead to reduced obesity [20]. While low-SES individuals in modern times tend to be more obese, the early 20th century was a time of an epidemiologic “nutrition transition” in the US [59], when higher-SES individuals were more likely to consume more obesogenic food, perhaps explaining our findings. Alternately, prior work suggests that education’s effects on reduced smoking may lead to increased obesity [60]. Future studies should replicate these findings in more recent cohorts as they age. Of note, estimates of the effects on total cholesterol and BMI were not robust to the adjustment of *p*-values for multiple hypothesis testing, so these results should be interpreted cautiously.

For most outcomes in the stress and inflammatory pathway, we were unable to rule out the null hypothesis that education had no effect; the exception was reduced depression in HRS. This may reflect improvements in coping or social support that result from increased education, or it may be due to changes in foundational skills like literacy [61]. For the null findings for the inflammatory outcomes, it may be that prior correlational studies suffered from confounding, e.g., due to difference in infectious exposures. It may also be that these analyses were underpowered, because sample sizes were smaller for biomarkers.

### Statistical considerations

The null associations in some IV models may be due to the larger sample size required for this type of analysis to attain comparable power relative to OLS models. For example, our analyses for telomere length were conducted on 3,500–5,000 participants in each data set. While meta-analysis does not produce stable estimates when combining only two effect estimates [62], future studies could consider conducting meta-analyses across additional data sets for these outcomes. Alternately, the null IV findings may suggest that some of these associations are confounded in OLS analyses by unobserved individual factors, and that IV models are able to better adjust for this bias.

Of note, none of the IV associations remained statistically significant when adjusting for state fixed effects. Several prior studies have demonstrated similar sensitivity of CSL IV models to the inclusion of fixed effects [27,63]. One possible explanation is that the observed associations may be confounded by other unobserved state-level policies or characteristics, such as labor market conditions. Alternatively, it may be that state fixed effects greatly reduce variation in the exposure, which hinges on state and year differences in CSLs, so that these models have limited statistical power. In nearly all cases, the confidence intervals for estimates from fixed effects models included both the IV estimates from models without fixed effects and the OLS estimates. The Durban-Wu-Hausman test we conducted suggests that fixed effects may not be warranted on empirical grounds, although there is disagreement on whether the Durban-Wu-Hausman test should be used to justify the omission of fixed effects. Ultimately, these inconsistencies imply that the results of our main models should be interpreted cautiously and replicated in future studies, although attaining sample sizes larger than those of this study will be challenging in the absence of meta-analyses.

### Strengths and limitations

This study has several strengths. It employed a natural experiment to produce rigorous estimates of the effects of education on CVD. It examined objectively measured biomarkers of disease in addition to self-reported health. Our examination of multiple outcomes allowed us to provide a more comprehensive picture of the mechanisms linking education and CVD. Additionally, analyses were replicated across two large nationally representative data sets, although differences in participant characteristics—e.g., NHANES was conducted during earlier years and included younger participants than HRS—means that estimates across the two studies may not be directly comparable in spite of adjustment for relevant sociodemographic variables and birth year.

In terms of limitations, self-reporting may have resulted in measurement error or reporting bias that could have been different by educational attainment, although biomarkers are not subject to this bias. Future studies could link diagnostic codes from death certificates or healthcare claims data to examine a wider scope of objective measures of disease. Second, a limitation of all IV analyses is the inability to test the assumption that no other factors confound the instrument-outcome association; here, state-level characteristics may influence both CSLs and CVD. We attempted to minimize this potential confounding by adjusting for state-level characteristics. IV analyses also only provide estimates of a “local average treatment effect” for individuals whose exposure is affected by the instrument, i.e., those who increased their educational attainment as a result of CSL implementation. This limits the generalizability of the resulting estimates, but these estimates also provide evidence on a specific policy to inform future interventions. Properly used, IV models tend to account for confounding more robustly than other observational techniques [64], although future studies could incorporate other forms of quasi-experimental or matching techniques that do not suffer from similar limitations. Additionally, findings may not generalize to the effects of education on CVD in the 21st century. This study may also be limited by selection bias, in that participants may be different from those who did not survive long enough to participate in HRS; our inclusion of younger participants from NHANES helps to strengthen our results. Relatedly, the use of linear rather than survival models is biased by differential follow-up time, because individuals with longer follow-ups are more likely to have the event. Unfortunately, NHANES is cross-sectional and does not include data on age of diagnosis, precluding us from carrying out survival models. Finally, while our study examines biomarkers that may be along the pathway linking education and CVD, future studies could undertake more formal mediation analyses to examine the direct and indirect effects through which education influences CVD, similar to prior studies examining other outcomes [10,11,61].

### Conclusions

This study employed quasi-experimental methods to provide rigorous estimates of the effect of education on CVD for the US context. Our findings support the established associations between education and reduced smoking, depression, and heart disease and improved HDL, suggesting that both health behaviors and stress are important mechanisms. Our study thereby contributes new knowledge on potential pathways through which education may influence CVD, and it adds to the evidence supporting broader implementation of interventions to target this key social determinant of health.

## Supporting information

S1 Analytic Plan Analytic plan. (PDF)

S1 STROBE Checklist STROBE checklist. (PDF)

S1 Text Supplemental methods. (PDF)

S1 Fig IV design. CSL, compulsory schooling law; IV, instrumental variable; SES, socioeconomic status.
(PDF)

## Abbreviations

CRP: C-reactive protein
CSL: compulsory schooling law
CVD: cardiovascular disease
HDL: high-density lipoprotein
HRS: Health and Retirement Study
IV: instrumental variable
LDL: low-density lipoprotein
NHANES: National Health and Nutrition Examination Survey
OLS: ordinary least squares
SES: socioeconomic status
