# Supplementary material for: Educational attainment and cardiovascular disease in the United States: A quasi-experimental instrumental variables analysis
Source: PLoS Med. 2019 Jun 25;16(6):e1002834. doi: 10.1371/journal.pmed.1002834 (PMC6592509; doi:10.1371/journal.pmed.1002834)
Supplement: S1 Analytic Plan — (PDF) [file pmed.1002834.s001.pdf]

Note: The following analytic plan was submitted to potential funders when we initially applied for funding to conduct this study. The main difference from the Methods described in the manuscript is that we added an additional wave of survey data (i.e., through 2012), which had become available prior to the onset of the analysis.

Methods

Data Sets and Sample Selection

This study relies on the integration and analysis of three large-scale national data sets. The first of three data sets employed by this study will be the 1980 U.S. Census 5% sample. As described below, we will be conducting a two-sample instrumental variables analysis, in which the first stage will be conducted using Census data. The resulting sample size is approximately 5 million individuals. This will allow for precise estimation of the first-stage coefficients in the IV analysis, i.e., the effect of CSLs on educational attainment.

The second data set we will employ is the HRS, a longitudinal study of individuals 50 years of age or older and their spouses. The first survey wave was collected in 1992, with biennial interviews through 2010. New cohorts were added in 1993, 1998, 2004, and 2010. Our sample includes U.S.-born individuals for whom there are data on state-of-birth and the health outcomes of interest. We will restrict our sample to individuals born before 1950, the years for which we have data on CSLs. Resulting sample sizes range from 10,000 individuals for biomarkers measured via blood tests, up to 25,000 individuals for anthropometric health outcomes such as blood pressure and weight.

The third data set is NHANES, a series of cross-sectional studies conducted biennially since 1999. We again restrict the sample to U.S.-born individuals born before 1950 for whom outcome data are available. This sample includes approximately 10,000 individuals.

Instruments

We have gathered data on CSLs to construct our two instruments. CSLs from 1906 to 1978 have been compiled previously (Acemoglu & Angrist, 1999; Glymour, Kawachi, Jencks, & Berkman, 2008; Lleras-Muney, 2002) using federal education reports usually available biennially. Data were collected on mandatory age at school enrollment, youngest age when it was legal to drop out of school, and youngest age when individuals could receive a work permit. For those years without data, we carried forward the most recently reported value of the state policy variable. For each respondent, years of compulsory schooling were calculated by taking the difference between enrollment age when respondents were 6 and minimum drop-out age when the respondents were 14. The second instrument is a continuous variable representing years of compulsory schooling for each individual, calculated by taking the difference between enrollment age when respondents were 6 and minimum work age when respondents were 14.

We assume that individuals remain in their state of birth until age 18. Prior studies have shown that cross-state migration was low during this period and that it was uncorrelated to the implementation of CSLs (Card & Krueger, 1990; Lleras-Muney, 2002).

Outcomes

The HRS and NHANES data sets include many health outcomes. We selected several biomarkers that have been used in prior work as objective markers of chronic disease and aging (Evans & Garthwaite, 2014; Jürges, Kruk, & Reinhold, 2013). These include anthropometric measures such as blood pressure and weight, which test the health behaviors pathway shown in Figure 1, as well as markers of inflammation and chronic disease collected via blood tests, which

capture the effects of the stress pathway. Unlike self-reported measures such as disease diagnoses, biomarkers are not dependent on prior access to healthcare and are not subject to reporting biases.

Control variables

In addition to controlling for individual-level factors (race, gender, birth year, and birth state), we also include variables representing characteristics of an individual's state of birth. These include per capita income, percentage black, urban, and foreign-born when the respondent was 6. These data were compiled by Glymour and Lleras-Muney using Statistical Abstracts of the United States (Glymour et al., 2008; Lleras-Muney, 2005). State characteristics were linearly interpolated for the years between reports.

Analytic Technique

We plan to use a two-sample instrumental variables (TSIV) analysis, a well-established method developed by Angrist and Krueger (1992). This is summarized in equations (1) and (2) (see Statistical Model, below). In the first stage of this two-stage analytic technique, educational attainment is predicted using the vector of instrumental variables described above and covariates C_k. To estimate the first stage, we will employ data from the 1980 U.S. Census 5% sample, in which questions about educational attainment are comparable to those asked in HRS and NHANES. These Census data have been used in previous studies using similar techniques (Angrist & Krueger, 1992; Glymour et al., 2008; Lleras-Muney, 2005). The predicted years of education from the first stage will then be used in the second stage as an independent variable to predict the health outcomes of interest. The education predictions from Census data will be linked to NHANES and HRS data by race, sex, birth state, and birth year.

TSIV is similar to the more commonly used two-stage least-squares method of IV analysis, except that the first and second stages are calculated using separate data sets. This allows for more precise estimation of equation (1), alleviating concerns of weak instrument bias resulting from instruments that explain only a small fraction of the variation in the endogenous variable (X). This method has been used previously in several studies examining the effects of CSLs (Angrist & Krueger, 1992; Glymour et al., 2008) and in other applications of IV analyses (Tchetgen Tchetgen, Walter, & Glymour, 2013).

Feasibility

We have conducted sample size calculations to ensure that our analyses will have a statistical power of 80% at an α of 5%. Depending on our estimate for the partial R-squared, the required sample size ranges from approximately 10,000 to 35,000. This will enable us to conduct analyses in the HRS and NHANES data sets separately for these outcomes, providing a cross-validation of our results. Also, preliminary analyses using the Census data indicated that CSLs were significantly associated with years of schooling. F-statistics, the most common measure of the strength of instruments, were well above 10, indicating that they are sufficiently strong for inclusion in the first stage.

STATISTICAL MODEL

As described in the Methods section above, our analytic technique relies on a two-sample instrumental variables methodology summarized by the following equations.

$$\text{Educ} = \beta_0 + \beta_1\text{Instrument} + \beta_2\text{C} + \varepsilon \quad (1)$$

$$\text{Health} = \gamma_0 + \gamma_1\widehat{\text{Educ}} + \gamma_2\text{C} + \varepsilon \quad (2)$$

Equation (1) represents the first stage of the TSIV analysis using data from the U.S. Census. Education is measured in years of schooling. Predicted education ($\widehat{\text{Educ}}$) from equation (1) will be used in equation (2), the second stage of the TSIV analysis. Equation (2) will be estimated using data from the HRS and NHANES. The primary coefficient of interest is γ_1, the improvement in health as a result of one additional year of schooling.

We will employ a vector of instruments described above. C is a vector of the individual- and state-level control variables included in both equations. β and γ represent the coefficients on the predictor variables of interest. ε is the error term.

REFERENCES

Acemoglu, D., & Angrist, J. D. (1999). *How large are the social returns to education? Evidence from compulsory schooling laws. Working Paper 7444*. Cambridge, Massachusetts: National Bureau of Economic Research.

Angrist, J. D., & Krueger, A. B. (1992). The effect of age at school entry on educational attainment: an application of instrumental variables with moments from two samples. *Journal of the American Statistical Association, 87*(418), 328-336.

Card, D., & Krueger, A. (1990). *Does school quality matter? Returns to education and the characteristics of public schools in the United States*: National Bureau of Economic Research.

Evans, W. N., & Garthwaite, C. L. (2014). Giving Mom a Break: The Impact of Higher EITC Payments on Maternal Health. *American Economic Journal: Economic Policy, 6*(2), 258-290.

Glymour, M. M., Kawachi, I., Jencks, C. S., & Berkman, L. F. (2008). Does childhood schooling affect old age memory or mental status? Using state schooling laws as natural experiments. *J Epidemiol Community Health, 62*(6), 532-537.

Jürges, H., Kruk, E., & Reinhold, S. (2013). The effect of compulsory schooling on health—evidence from biomarkers. *Journal of population economics, 26*(2), 645-672.

Lleras-Muney, A. (2005). The Relationship Between Education and Adult Mortality in the United States. *The Review of Economic Studies, 72*(1), 189-221.

Lleras-Muney, A. (2002). Were Compulsory Attendance and Child Labor Laws Effective? An Analysis from 1915 to 1939. *Journal of Law and Economics, 45*(2), 401-435.

Tchetgen Tchetgen, E. J., Walter, S., & Glymour, M. M. (2013). Commentary: Building an evidence base for mendelian randomization studies: assessing the validity and strength of proposed genetic instrumental variables. *International Journal of Epidemiology, 42*(1), 328-331.
