# Supplementary material for: Educational attainment and cardiovascular disease in the United States: A quasi-experimental instrumental variables analysis
Source: PLoS Med. 2019 Jun 25;16(6):e1002834. doi: 10.1371/journal.pmed.1002834 (PMC6592509; doi:10.1371/journal.pmed.1002834)
Supplement: S1 Text — (PDF) [file pmed.1002834.s003.pdf]

SUPPLEMENTAL METHODS

OLS Analysis

OLS models are represented by the following equation:

$$Health_{ist} = \gamma_0 + \gamma_1 Educ_{ist} + \gamma_2 X_{ist} + \gamma_3 S_{st} + \gamma_4 \theta_t + \varepsilon_{ist}$$

Here, $Health$ is a given health outcome of interest for individual i born in state s in year t, $Educ$ is the individual's self-reported educational attainment, X is a vector of individual-level covariates, S is a vector of state-level time-varying covariates, θ represents year-of-birth fixed effects, and ε represents robust standard errors clustered by state of birth. We present the results of linear models for both continuous and binary outcomes to allow for comparability in the reporting of effect estimates as beta coefficients, although logistic models for binary outcomes were similar in magnitude and statistical significance (data available upon request).

IV Analysis

IV analysis rests on the assumption that there is not a separate pathway linking the instrument and outcome that does not pass through the predictor. In this case, there are unlikely to be plausible pathways linking CSLs to health other than through duration of educational attainment. For example, one prior study suggests that school quality is unlikely to be affected by changes in CSLs [2].

Another assumption is that there are no confounders of the relationship between the instrument and the outcome; for example, there may be concern that other state-level factors may confound the relationship between CSLs and health [3]. We address this with the inclusion of additional variables representing state-level characteristics, a technique used in prior work [4-7], as well as secondary models including state fixed effects.

For years without data, the most recently reported value of the state CSL variable was carried forward. We assume that individuals remained in their state of birth until age 18; prior studies have shown that cross-state migration was low during this period and that it was uncorrelated to the implementation of CSLs, so any measurement error would bias our results to the null [8,9].

The IV models employed in this study can be represented by the following two equations:

$$Educ_{ist} = \alpha_0 + \alpha_1 CSL_{st} + \alpha_2 X_{ist} + \alpha_3 S_{st} + \alpha_4 \theta_t + \varepsilon_{ist} \quad (1)$$

$$Health_{ist} = \beta_0 + \beta_1 \widehat{Educ}_{ist} + \beta_2 X_{ist} + \beta_3 S_{st} + \beta_4 \theta_t + \upsilon_{ist} \quad (2)$$

Equation (1) represents the first stage of the IV analysis, in which education, the endogenous variable, is regressed on the two instruments representing years of compulsory schooling, while adjusting for individual- (X) and state-level (S) characteristics as well as fixed effects for year θ. Using the coefficients from the first stage, a predicted level of education is produced for every individual in the sample. In this study, the first stage was conducted in the Census sample, and predicted education was linked to individuals in HRS and NHANES by gender, race, year of birth, and state of birth. This predicted education was then used in Equation (2) with health as the dependent variable, which represents the second stage of the IV analysis. The coefficient of interest is β_1, which represents the causal effect of an additional year of educational attainment on the health outcome of interest. Robust standard errors υ were clustered by state of birth. This type of IV analysis is known as two-sample IV (TSIV) analyses, which is an extension of the more commonly used two-stage least squares (2SLS) IV analyses.

Using a two-sample approach allowed for more precise estimation of the first stage as the Census sample size is much larger, thereby alleviating concerns of weak instrument bias resulting from instruments that explain only a small fraction of the variation in the endogenous variable. In this case, for example, the F-statistic for the first stage using HRS data was 11.2,

while the F-statistic using the Census sample was 793.7. TSIV is also useful in situations where a single sample does not contain all three of the instrument, predictor, and outcome. In this case, NHANES does not contain a variable for continuous years of education, such that the first stage could not be carried out, thereby necessitating the TSIV approach we employ here.

Standard errors were calculated using a bootstrapping technique. Using 100 bootstrapped samples from the Census, we produced 100 values of predicted education for each individual (Equation 1). Each of these values for predicted education was then used to estimate β_1 (Equation 2) in 100 bootstrapped samples in HRS and NHANES, producing $100 \times 100 = 10{,}000$ estimates. The estimates reported here represent the mean of these 10,000 estimates of β_1, and 95% confidence intervals are the estimates at the 2.5^{th} and 97.5^{th} percentile. HRS and NHANES survey weights were not employed due to our use of the bootstrapping technique to calculate standard errors, and since the utility of weighting is diminished when the goal of inference is determining causal effects rather than population estimates [10].

In Census data, an additional year of compulsory schooling using the first instrument—difference between enrollment and dropout ages—led to an increase in educational attainment of 0.27 years (95% CI: 0.26, 0.28). For the second instrument—the difference between enrollment and minimum work ages—an additional year of schooling led to an increase in educational attainment of 0.21 years (95% CI: 0.20, 0.22). This satisfied the IV assumption that the two instruments were each associated with the endogenous variable of interest. This supports the use of the Census sample for the first stage, given its larger size and subsequently greater precision in estimating the first stage.

SUPPLEMENTAL REFERENCES

1. Von Hippel PT Regression with missing Ys: An improved strategy for analyzing multiply imputed data. Sociological Methodology 2007; 37: 83-117.
2. Brunello G, Fort M, Weber G, Weiss CT Testing the internal validity of compulsory school reforms as instrument for years of schooling. Available at SSRN 2346850 2013.
3. Edwards LN An Empirical Analysis of Compulsory Schooling Legislation, 1940-1960. Journal of Law and Economics 1978; 21: 203-222.
4. Fletcher J New evidence of the effects of education on health in the US: Compulsory schooling laws revisited. Social Science & Medicine 2015; 127: 101-107.
5. Glymour MM, Kawachi I, Jencks CS, Berkman LF Does childhood schooling affect old age memory or mental status? Using state schooling laws as natural experiments. J Epidemiol Community Health 2008; 62: 532-537.
6. Lleras-Muney A The Relationship Between Education and Adult Mortality in the United States. The Review of Economic Studies 2005; 72: 189-221.
7. Nguyen T, Tchetgen Tchetgen E, Kawachi I, Gilman S, Walter S, Liu S, et al. Instrumental variable approaches to identifying the causal effect of educational attainment on dementia risk. Annals of Epidemiology 2016; 26: 71-76.
8. Card D, Krueger A Does school quality matter? Returns to education and the characteristics of public schools in the United States. The Journal of Political Economy 1992; 100: 1-40.
9. Lleras-Muney A Were Compulsory Attendance and Child Labor Laws Effective? An Analysis from 1915 to 1939. Journal of Law and Economics 2002; 45: 401-435.
10. Solon G, Haider SJ, Wooldridge JM What Are We Weighting For? Journal of Human Resources 2015; 50: 301-316.
