# Supplementary material for: Educational attainment and cardiovascular disease in the United States: A quasi-experimental instrumental variables analysis
Source: PLoS Med. 2019 Jun 25;16(6):e1002834. doi: 10.1371/journal.pmed.1002834 (PMC6592509; doi:10.1371/journal.pmed.1002834)
Supplement: S1 Fig — CSL, compulsory schooling law; IV, instrumental variable; SES, socioeconomic status. (PDF) [file pmed.1002834.s004.pdf]

S1 Fig. IV design

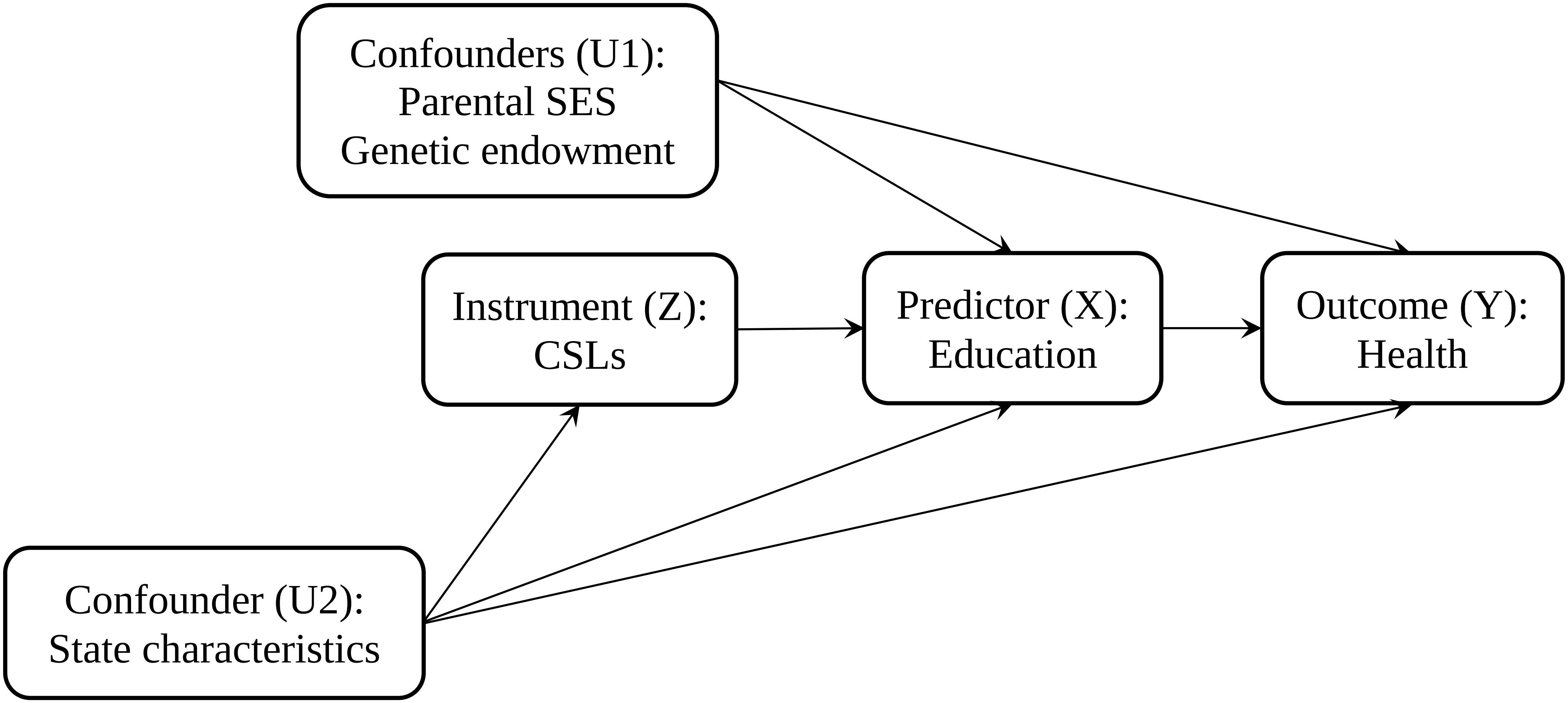

Note: CSL = compulsory schooling laws; IV = instrumental variables; SES = socioeconomic status
